# Functional engagement of white matter in resting-state brain networks

**DOI:** 10.1016/j.neuroimage.2020.117096

**Published:** 2020-06-26

**Authors:** Muwei Li, Yurui Gao, Fei Gao, Adam W. Anderson, Zhaohua Ding, John C. Gore

**Affiliations:** a Vanderbilt University Institute of Imaging Science, 1161 21st Ave. S, Medical Center North, AA-1105, Nashville, TN, 37232, USA; b Department of Radiology and Radiological Sciences, Vanderbilt University Medical Center, 1161 21st Ave. S, Medical Center North, Nashville, TN, 37232, USA; c Department of Biomedical Engineering, Vanderbilt University, 2301 Vanderbilt Place, Nashville, TN, 37235, USA; d Department of Electrical Engineering and Computer Science, Vanderbilt University, 2301 Vanderbilt Place, Nashville, TN, 37235, USA; e Shandong Medical Imaging Research Institute, Shandong University, Jinan, China

**Keywords:** Resting-state, Brain network, White matter, Partial correlation, Engagement map, Physiological noise

## Abstract

The topological characteristics of functional networks, derived from measurements of resting-state connectivity in gray matter (GM), are associated with individual cognitive abilities or specific dysfunctions. However, blood oxygen level-dependent (BOLD) signals in white matter (WM) are usually ignored or even regressed out as nuisance factors in the data analyses that underlie network models. Recent studies have demonstrated reliable detection of WM BOLD signals and imply these reflect associated neural activities. Here we evaluate quantitatively the contributions of individual WM voxels to the identification of functional networks, which we term their engagement (or conceptually, their importance). We quantify the engagement by measuring the reductions of connectivity, produced by ignoring the signal fluctuations within each WM voxel, with respect to both the entire network (global) or a single GM node (local). We observed highly reproducible spatial distributions of global engagement maps, as well as a trend toward increased relevance of deep WM voxels at delayed times. Local engagement maps exhibit homogeneous spatial distributions with respect to internal nodes that constitute a well-recognized sub-functional network, but inhomogeneous distributions with respect to other nodes. WM voxels show distinct distributions of engagement depending on their anatomical locations. These findings demonstrate the important role of WM in network modeling, thus supporting the need for changes of conventional views that WM signal variations represent only physiological noise.

## 1. Introduction

Spontaneous low-frequency fluctuations in blood oxygen level-dependent (BOLD) signals, measured by functional magnetic resonance imaging (fMRI), are considered to be coupled to intrinsic neural activity in a resting brain (Fox and Raichle, 2007). The synchronous variations of BOLD signals from separate cortical sites are similarly interpreted as reflecting functional connectivities, independent of the identification of any anatomical connections between them (Biswal et al., 1995). Measuring the pair-wise connectivities of segregated regions in gray matter (GM) throughout the brain provides a mathematical approach that allows the derivation of functional networks, which in turn form the basis of current models of the functional organization of the brain (Fair et al., 2008; Md et al., 2003; Micheloyannis et al., 2009; Supekar et al., 2009). In the pursuit of identifying such networks, white matter (WM), though making up half of the brain by volume, has been consistently overlooked in resting-state studies. In fact, the average BOLD fluctuations in WM have often been considered as a nuisance covariate to be removed by regression in order to reduce physiological noise while identifying each network (Behzadi et al., 2007; Caballero-Gaudes and Reynolds, 2017). In recent years, however, a growing number of studies have consistently demonstrated that BOLD signals within WM appear to reflect neural activity (Ding et al., 2018, 2016; 2013; Ji et al., 2017). For example, our previous work observed that WM fluctuations may be strongly correlated with signals in specific GM volumes during rest, and these correlations can be modulated by specific functional demands (Ding et al., 2018). Peer et al. identified an intrinsic functional organization of WM networks using a clustering approach based on resting-state data (Peer et al., 2017). This work highlighted the potential functional role of WM networks because of their close similarity with resting-state GM networks. Li and colleagues identified weak small-world topology and non-random modularity in WM functional networks (Li et al., 2019a). Marussich et al. (2017) used independent components analysis (ICA) to derive spatially independent patterns within WM that appeared to reorganize during natural vision. These and other relevant contributions were included in a recent Perspective by Grajauskas et al. (2019) who argue strongly that WM activity should not be treated as a nuisance regressor but instead be recognized as important in analyses of brain function. Moreover, an increasing number of studies have reported that alterations of WM networks are associated with brain disorders including schizophrenia, Parkinson’s disease and epilepsy (; Ji et al., 2019; Jiang et al., 2019, 2018). Taken together, these findings indicate that WM-GM and WM-WM correlations reveal an underlying organization and suggest further investigations are justified of the possible contributions of WM signals to the identification of well-established brain networks that by convention consist of GM alone.

In graph theory, a functional network is typically composed of a set of nodes and edges (Wang, 2010), where a node corresponds to a region-of-interest (ROI), a group of voxels that are considered anatomically or functionally homogeneous, while an edge represents the link between nodes. The connections (edges) are typically quantified by metrics such as full correlation (i.e. Pearson’s correlation coefficient) (Friston, 2011) or partial correlation (Marrelec et al., 2006). Compared to partial correlations, which estimate only direct connections, the full correlation may reflect only a marginal association because it includes both direct and indirect connections between two nodes (Marrelec et al., 2006). For example, an indirect connection between node *A* and *C*, which might be caused by their shared connections to node *B*, can be eliminated by using the partial correlation which regresses out confounding connections between *A* and *B* or *B* and *C* during the calculation (Smith et al., 2011). As such, partial correlation often identifies a reduced number of edges and consequently leads to simpler network topologies when compared with full correlation (Liang et al., 2012). These findings imply that when a node that strongly exerts an influence on other nodes, i.e., a hub (Jonathan D. Power et al., 2013), is regressed out by partial correlation, the network may reorganize its topology and exhibit lower efficiency in communications between the residual nodes than that when ruling out a node that exchanges information less broadly with the rest. The magnitude of the reduction in connectivity caused by the absence of a node thus encodes the importance or engagement of that node as a waystation supporting connections over the network. This concept of engagement provides a convenient framework for examining the contributions of WM signals by quantifying to what extent a network would be affected by removal of WM BOLD signals.

One simple approach to quantifying the engagement of WM voxels would be to perform a partial correlation between identified GM nodes while controlling for the time series of WM units (voxels or ROIs), and to measure the reductions in connectivities, which then reflect the engagement of the WM. However, partial correlation with a brute-force ruling out of WM fluctuations falsely assumes a precise synchronization of spontaneous BOLD signals in WM and GM, which can result in decreases in underlying WM-GM correlations and thus underestimate the importance of the WM. The delayed responses of BOLD signals in WM have been reported in several previous studies (Erdoğan et al., 2016; Thomas et al., 2014; Tong et al., 2017). For example, our previous study observed that BOLD signal waveforms in stimulus-relevant WM pathways are synchronous with the applied stimuli but show various degrees of time delay (Ding et al., 2018). Similar findings have also been reported in cerebrovascular reactivity (CVR) studies during hypercapnic challenges (Bhogal et al., 2015; Blockley et al., 2011; Poublanc et al., 2015; Thomas et al., 2014), where WM responses showed a temporal delay compared with GM, which was attributed to the longer time it takes for extravascular CO2 to change. The delayed responses observed in WM BOLD signals are likely mainly due to differences in the WM hemodynamic response function (HRF) which depends on the local neurovascular coupling and whose latency reflects the lower vascular density and longer distances between feeding arterioles and areas of increased oxygen use within WM. Though particularly relevant for studies involving a task, the notion of a delayed HRF is relevant to resting-state data as well. Thus before partial correlations can be legitimately performed, allowances need to be made for the possibility that a WM signal time series reflects synchronous activity some time delayed relative to correlated signals in GM. In addition, our recent study shows that the onset of WM HRF varies as a function of WM location (Li et al., 2019b). Taking this into account, here we perform our analyses piecewise, with successively increasing delays that span physiologically realistic ranges throughout the brain. A major goal of the current study is to identify the spatial distributions of the engagement of WM voxels in brain networks, as well as how these distributions vary with time, thus allowing for a better understanding of the functional contributions of WM to connectivity measures within well-established brain networks.

## 2. Materials and methods

### 2.1. Subjects

The present study was approved by the Vanderbilt University Institutional Review Board. Written informed consent was obtained from each subject. Fifty-three healthy individuals (22M/31F; age, 34.7 ± 11.2 yrs) with no histories of neurological or psychiatric disorders were included. Patient information was confirmed for the second time by an MRI technician before the scanning through a prescreening survey.

### 2.2. Imaging

Images were acquired using a 3T MRI scanner (Philips Healthcare, Best, Netherlands) installed at Vanderbilt University Institute of Imaging Science. Each subject was scanned in a supine, head-first position with restricting pads placed within the 32-channel head coil to ensure stability. Resting state fMRI images were acquired with TR 2 s, TE 35 ms, SENSE factor 2, matrix size = 80 × 80,FOV = 240 × 240 mm^2^, 34 slices 4mm thickness a 0.5 mm gap, and 200 dynamics. During each scan, the subjects were instructed to stay awake with their eyes closed, remain still, and think of nothing. As anatomical references, high-resolution T1-weighted images were acquired using a three-dimension (3D) magnetization-prepared rapid gradient echo (MP-RAGE) sequence at a voxel size of 1× 1 ×1 mm^3^.

### 2.3. Preprocessing

fMRI images were preprocessed using the statistical parametric mapping (SPM12) software (Penny et al., 2011). First, the images were corrected for intra-scan acquisition time differences and inter-scan head motions. Second, for each subject, probabilistic masks (range from 0 to 1) depicting GM, WM, and cerebrospinal fluid (CSF) were obtained by segmenting the T1 structural images. Next, these masks, as well as T1 images, were co-registered to the native space of fMRI images for the same subject. All resulting images, including the fMRI, T1, and GM/WM/CSF masks, were then spatially normalized to a standard space (Montreal Neurological Institute (MNI)) coordinates (Evans et al., 1992), at a voxel size of 1.5 × 1.5 × 1.5 mm^3^. The covariates from the head motion were regressed out from the time-series using Friston’s 24-parameter model (Friston et al., 1996). Confounding effects of physiological fluctuations, such as cardiac pulsations and respiration-induced modulations, were removed using a CompCor approach (Behzadi et al., 2007). Specifically, nuisance variables were derived from the anatomical noise ROI, identified within cerebrospinal fluid (CSF), and were subsequently regressed out from the BOLD time series. Note that, we also included the voxel-wise correlation analysis of WM signals (before regression) with the head movement and physiological measures in the supplementary file (Figure S1). Finally, the voxel-wise time courses extracted from the normalized fMRI images were corrected for signal drifts, temporally filtered with a bandpass filter (0.01–0.1 Hz), and normalized to zero mean and unit variance. Before filtering, the fractional amplitude of low-frequency fluctuations (fALFF), which stands for the ratio of the power spectrum at low-frequency (0.01–0.1 Hz) to that of the entire frequency range, was calculated for each time-series.

### 2.4. WM engagement maps

**Fig. 1.**
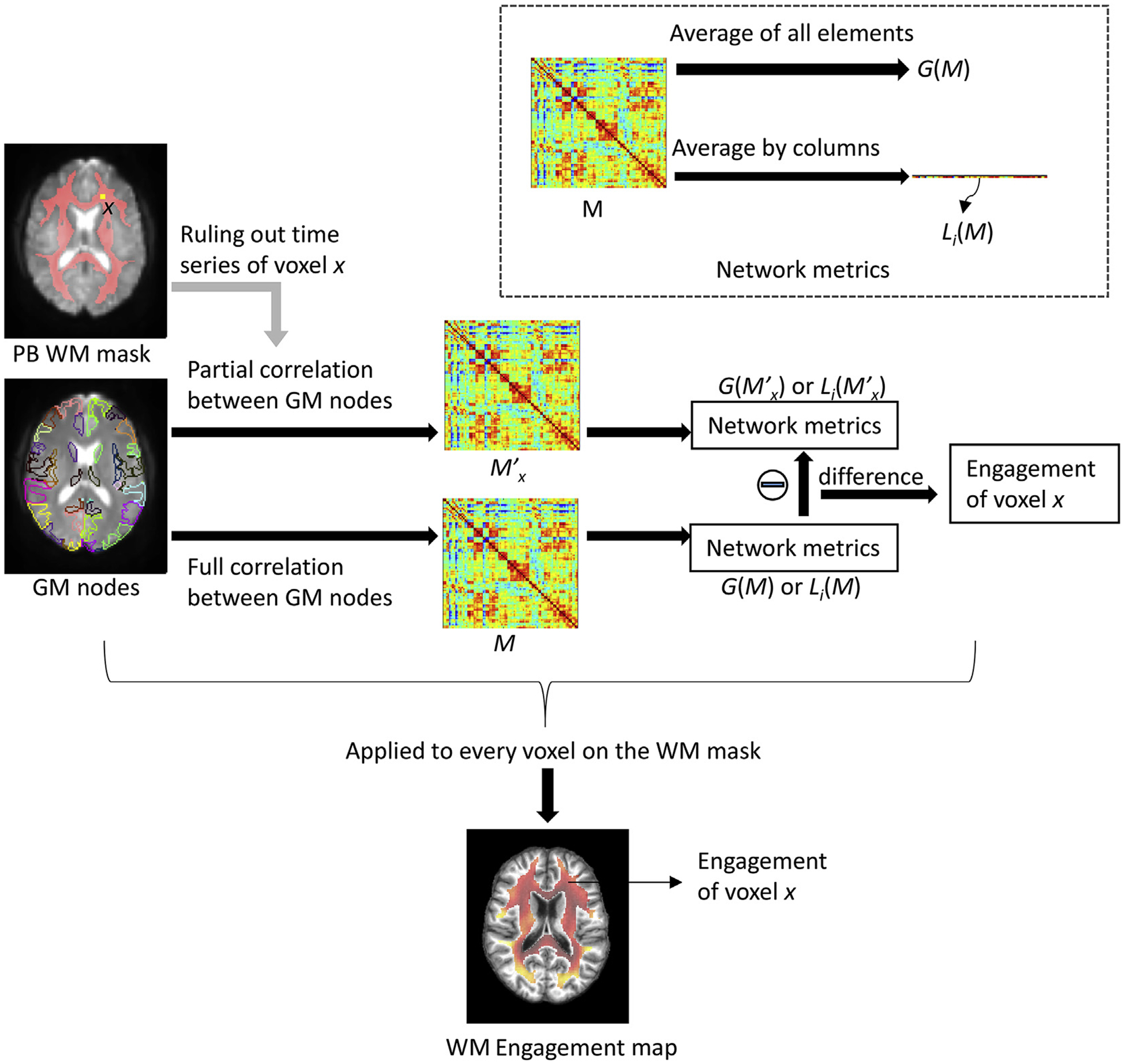
Flowchart of the derivation of WM engagement map for a single individual.

The overall pipeline of the derivation of WM engagement maps is illustrated in Fig. 1. It starts with the definition of a population-based (PB) WM mask, a group of voxels whose functional engagement is to be evaluated. Specifically, the normalized probabilistic WM masks were averaged across all subjects and then binarized with a tight threshold (>0.95) to provide, with high confidence, a set of voxels that were anatomically designated as WM with minimal contamination by partial volume effects from adjacent GM. Meanwhile, a PB averaged GM mask was generated through a similar process but using the normalized probabilistic GM masks and a relatively loose threshold (>0.6) for including as many GM voxels as possible in the mask. The definition of GM nodes could then be obtained by multiplying the PB GM mask with the Automated Anatomical Labeling (AAL) atlas (Tzourio-Mazoyer et al., 2002) which parcellated the GM into 90 macroscopic brain structures in MNI space. Temporal correlations among all pair-wise nodes produced both a full connectivity matrix *M*, and a partial connectivity matrix *M’*_*x*_ in which the time-series of a WM voxel *x* was controlled. A global network connectivity metric *G*(*M*) was calculated by summing up all pair-wise connectivities. Local metrics {*L*_*i*_(*M*), *i* = 1,2,3, …, 90} were calculated by adding the connectivity values between a node *i* and all other nodes for each of the 90 GM nodes. These metrics were obtained for both *M* and *M’*_*x*_. The difference between *G*(*M*) and *G*(*M’*_*x*_) represents the global functional engagement of WM voxel *x* with respect to the entire network, while the difference between each *L*_*i*_(*M*) and *L*_*i*_(*M’*_*x*_) measures the local engagement of *x* in mediating a sub-circuit centroid at node *i*. Then, for each individual, values of the engagement map were rescaled to the percentage difference from mean of the map. The purpose of this rescaling, for one thing, is to give the value of the engagement map a unit. For another, it shifts the mean of the map to zero thereby removing inter-individual differences in the global scales. This is similar to the analysis of ALFF (amplitude of low-frequency fluctuation) introduced by Yu-Feng et al. (2007). Finally, the group-based engagement maps were generated using a one-sample *t*-test over the fifty-three individual maps. Voxels beyond a threshold (voxel-wise inference, p < 0.01, FDR corrected) are considered significantly engaged. In practice, any threshold can be applied according to different needs in data interpretation. Here we use p < 0.01 (FDR corrected) for better visualization of the most engaged voxels.

During partial correlation estimates, the time-series of a controlled voxel *x* was shifted forward by *t* s (*t* = 0, 2, 4, 6) to compensate for a *t-*second delay of WM signals relative to GM. Thus the engagement map associated with each *t* s delay represents the distribution of the voxel’s engagement at a time point *t* seconds after the network modeling. In addition, the time-series of voxel *x* was replaced by the average signal of the 5 × 5 × 5 WM voxels neighboring voxel *x* so as to improve the signal to noise ratio (SNR). Note that if the neighborhood contains non-WM voxels according to the PB WM mask, these voxels were not included in the average.

### 2.5. Evaluation of global engagement on different depths of WM

**Fig. 2.**
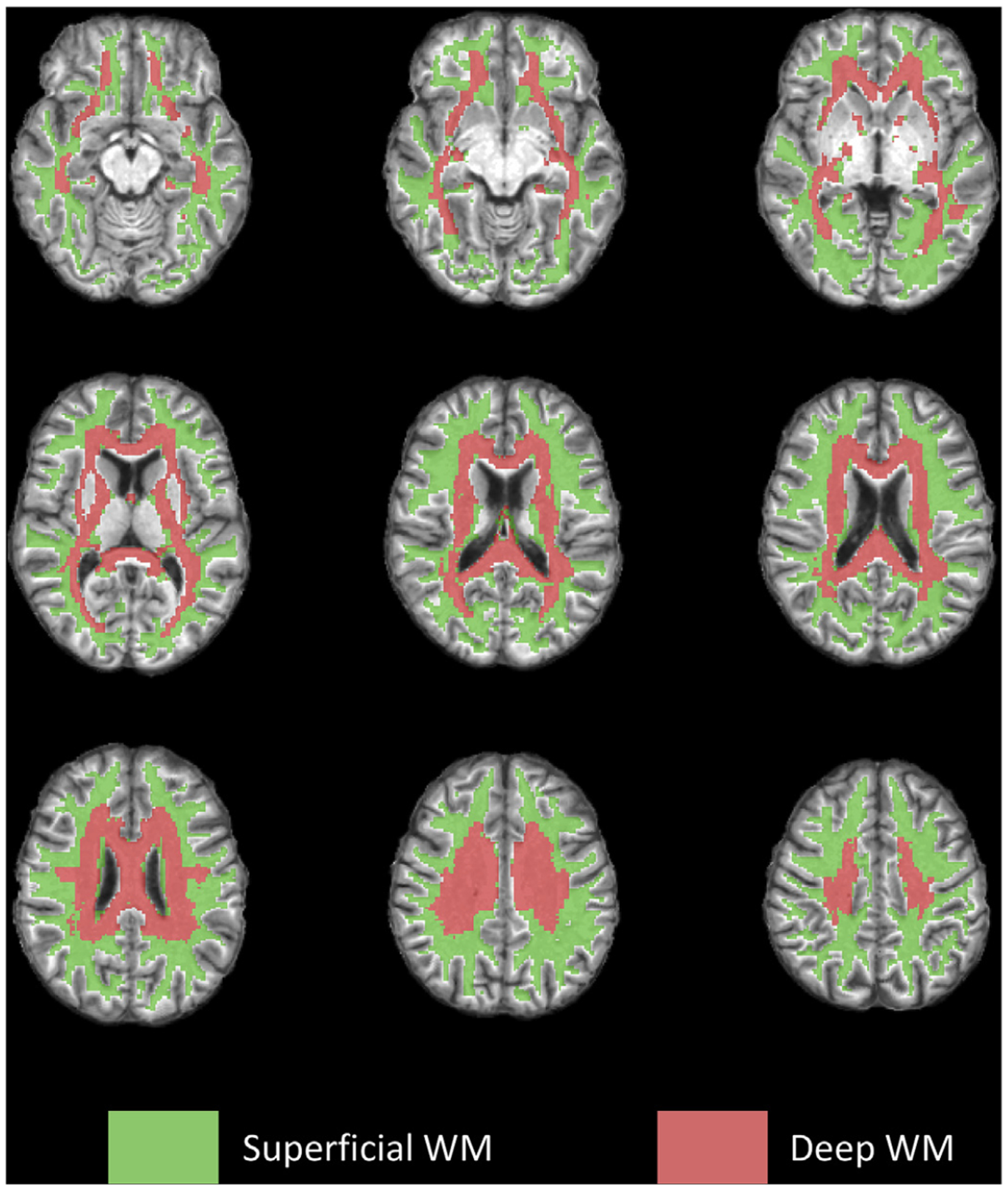
Spatial distribution of superficial and deep WM voxels.

The global functional engagement was evaluated separately for superficial WM and deep WM voxels. A second PB WM mask was created by setting a looser threshold of 0.5 to retain sub-cortical WM voxels. Within this mask, those voxels that overlapped with the deep WM structures that are manually labeled in the JHU-DTI-MNI atlas (Oishi et al., 2009) were designated as deep WM (see Fig. 2), while the rest were considered superficial WM voxels.

### 2.6. Evaluation of reproducibility of global functional engagement maps

The fifty-three subjects were randomly divided into two even groups (26 vs. 27). The average global engagement for each group was calculated when assuming 0, 2, 4, 6 s delay separately. The correlation coefficients between the global engagement maps of the two groups were also calculated.

### 2.7. Comparison between global functional engagement and fALFF

**Fig. 3.**
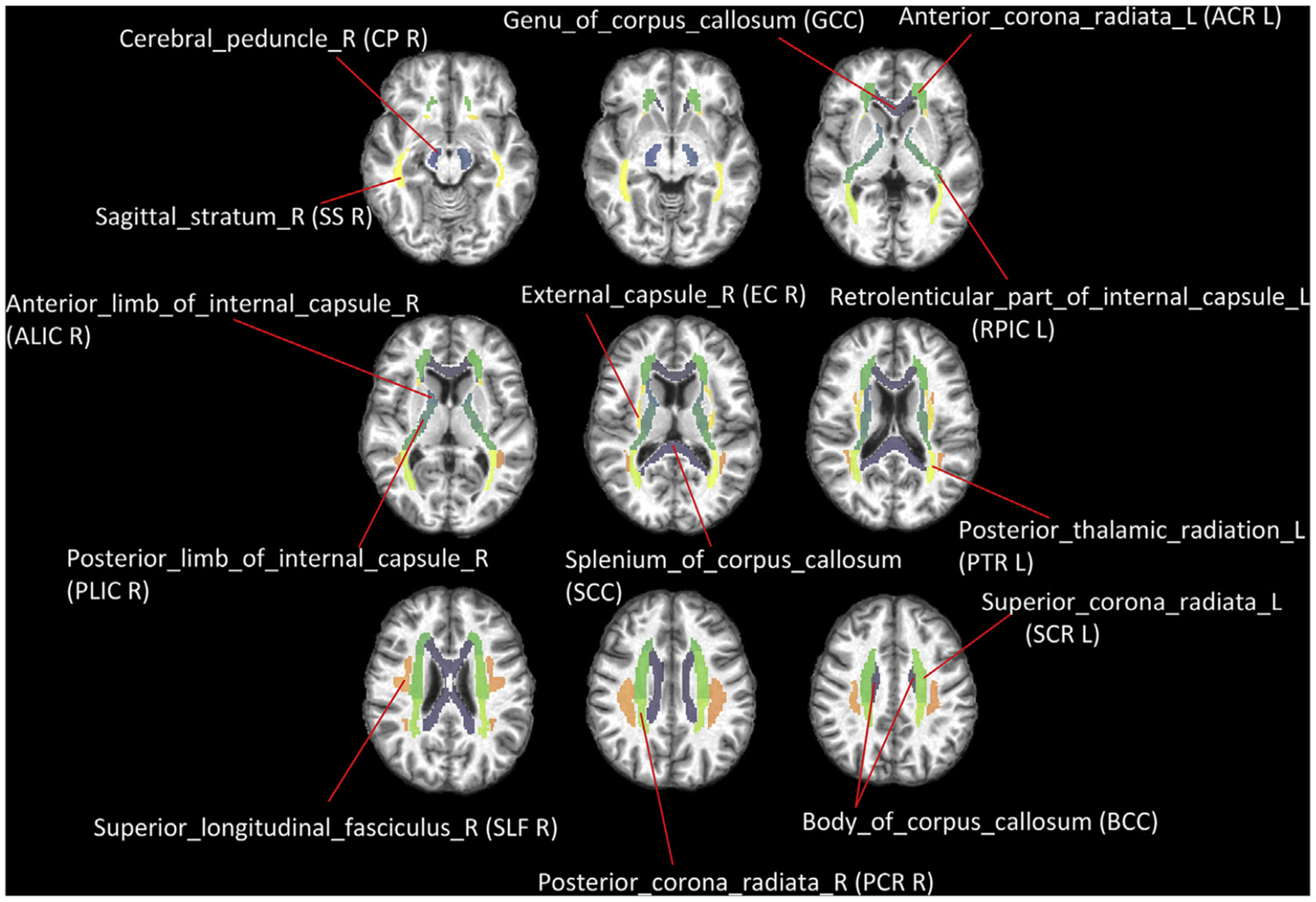
Definition of selected WM tracts based on JHU-MNI-ss atlas.

We first parcellate the PB WM mask into 25 anatomical structures, termed as tracts, according to the JHU-DTI-MNI atlas as shown in Fig. 3. The average fALFF was calculated for each tract. At a global level, the number of significantly engaged (p < 0.01, FDR corrected) voxels that overlap with each of 25 WM tracts is measured. Its relationship to the average fALFF value of the tract is evaluated using Pearson’s correlation. Note that there are 48 structures originally defined in the JHU atlas. However, 23 of them are small in size and too close to gray matter and so not covered by the WM mask created by thresholding the probabilistic WM segmentation with a very high threshold (0.95). Therefore they are not included in the analysis.

### 2.8. Evaluation of local engagement

According to the pipeline, local functional engagement maps for every GM node can be calculated. Here we focus on two well-recognized functional networks: the visual network (Thomas Yeo et al., 2011), which here is represented as a single functional node (visual cortex, defined by area 17 in Broadmann atlas) and the default mode network (DMN) (Bressler and Menon, 2010) which is composed of three major interconnected nodes including angular gyrus (AG), medial prefrontal cortex (mPFC) and posterior cingulate cortex (PCC). We evaluated the local functional engagement associated with each of these four GM nodes in terms of the reduced amounts of average connectivity between the investigated nodes and all other GM nodes when ruling out the BOLD signal in each voxel *x*.

To understand the spatial distribution of local functional engagement, we first parcellate the PB WM mask into 25 anatomical structures, termed as tracts, according to JHU-DTI-MNI atlas as shown in Fig. 3. Then the number of significantly engaged (p < 0.01, FDR corrected) voxels that are overlapped with each of 25 WM tracts was quantified.

## 3. Results

### 3.1. Group-based global functional engagement of WM

**Fig. 4.**
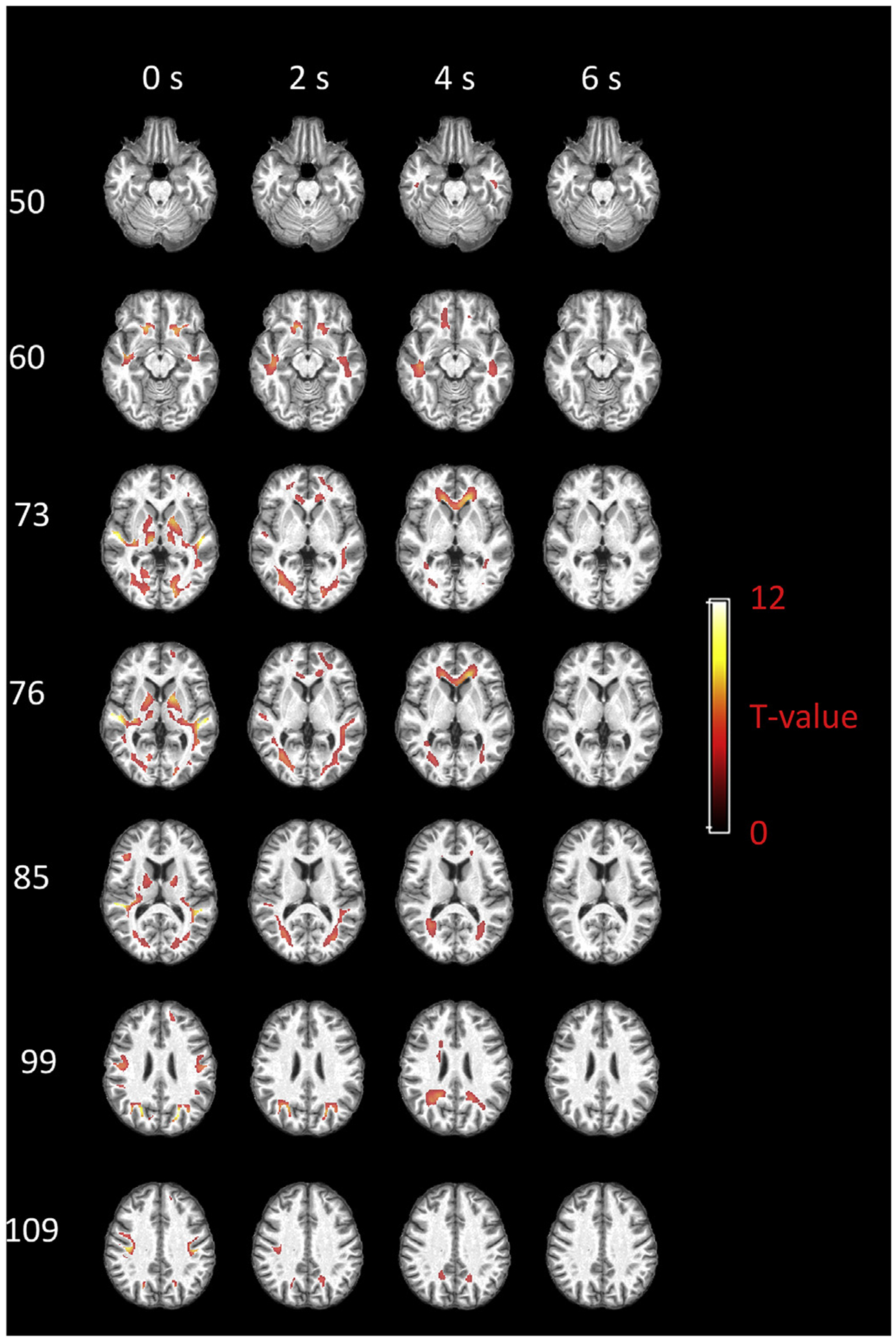
Group-based global functional engagement map of WM. Selected axial slices displayed in rows and the maps regarding four assumptions of WM delays displayed in columns. T values beyond 3.25 (p < 0.01, FDR corrected) are shown.

The group-based global functional engagement of each voxel within the PB WM mask is shown in Fig. 4, with selected axial slices displayed in rows and the maps obtained using four different assumptions of WM delays displayed in columns. When no delay is assumed for WM, the significantly engaged voxels are mainly distributed in subcortical WM areas that are adjacent to fronto-orbital gyrus, superior/middle temporal gyrus, superior/middle occipital gyrus, lingual gyrus, pre/post-central gyrus, as well as in WM areas that are adjacent to deep gray matter nuclei including putamen, caudate and thalamus. By assuming a 2 s delay, the distribution changes, with more engaged voxels identified in deep WM, e.g., anterior coronal radiation, posterior thalamic radiation, and sagittal stratum. Some of the subcortical voxels still show high engagement at this delay. When a 4 s delay is assumed, anterior coronal radiation still shows high engagement. Deeper WM areas, including genu of corpus callosum and splenium of corpus callosum, are identified with high engagement as well. Meanwhile, less important voxels can be found within subcortical WM areas. When assuming a 6 s delay, most of the WM engagement disappears.

### 3.2. Global functional engagement for WM at different depths

**Fig. 5.**
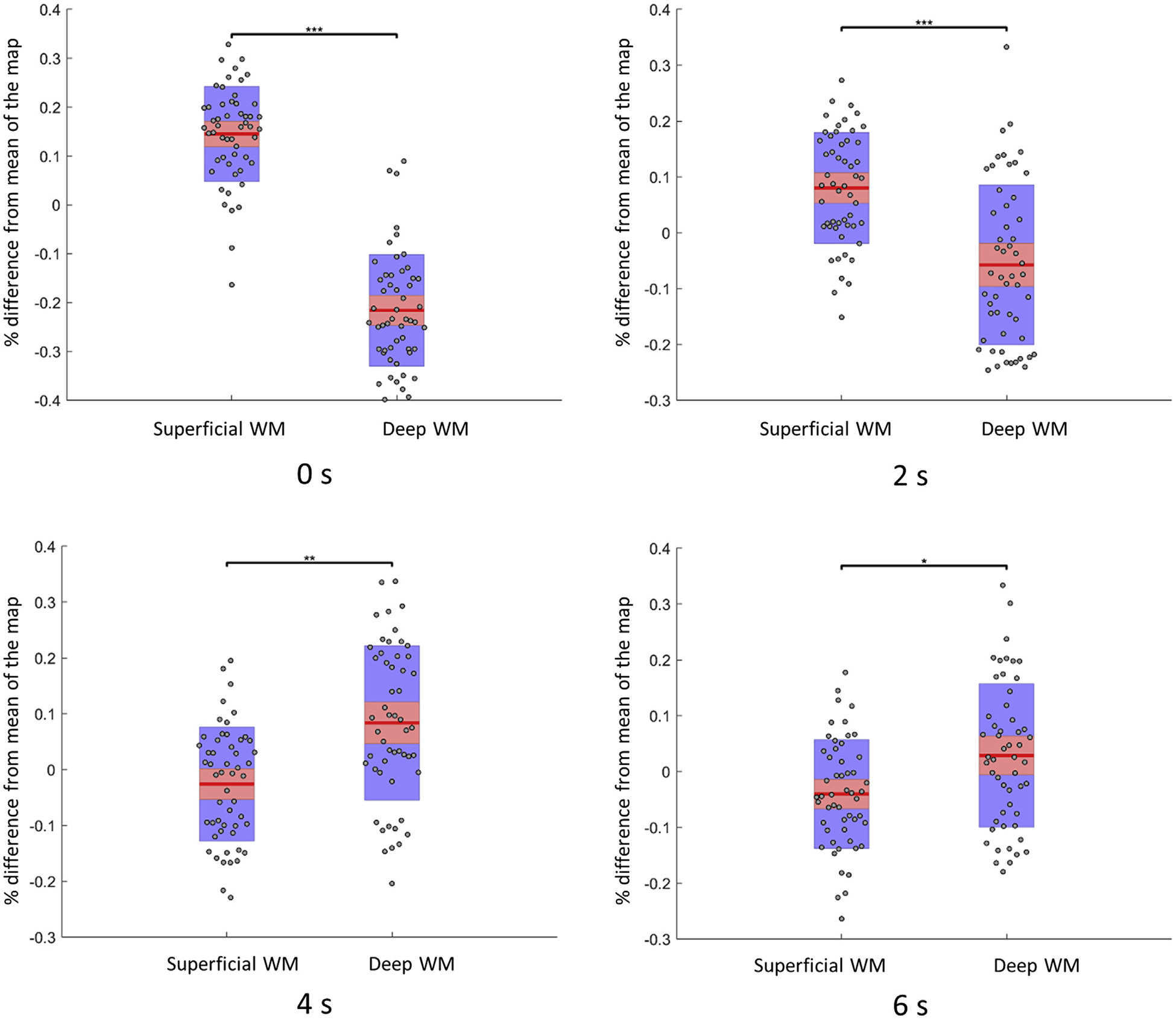
Global functional engagements in superficial WM and deep WM, and their changes along time. The y-axis is the global engagements of twenty-two subjects. Each point denotes averaged percentage change to mean within superficial or deep WM mask. Points are laid over a 1.96 SEM (95% confidence interval) in red, a 1 SD in blue and a center line that denotes the median. *** denotes p < 0.001, * denotes p < 0.05.

Fig. 5 shows the global functional engagement values for superficial and deep WM. Superficial WM exhibits significantly higher engagement across subjects (*P* < 0.001) compared to deep WM when assuming a 0 s delay for WM. This difference remains significant but decreases (*P* < 0.001) assuming a 2 s delay. With 4 s delay, deep WM shows significantly higher engagement (*P* < 0.001) compared to the superficial WM. This significant difference disappears when assuming a 6 s delay for WM.

### 3.3. Reproducibility of global functional engagement maps

**Fig. 6.**
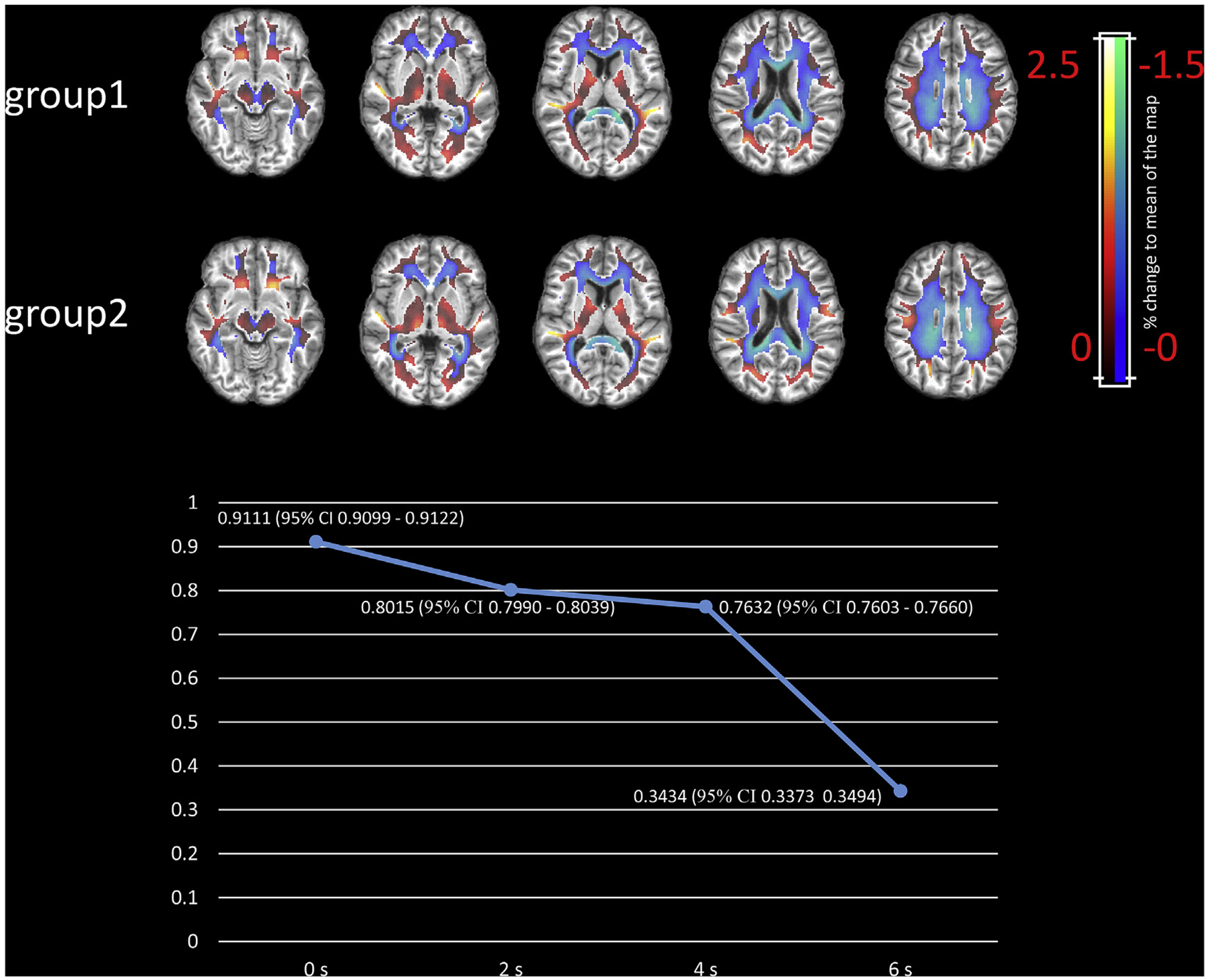
Reproducibility of global functional engagement maps. The upper panel: global engagement maps (0 s delay) of two even groups. The lower panel: correlation coefficients between global engagement maps of the two groups when assuming different delays.

To evaluate the reproducibility of global functional engagement maps, we randomly divided the twenty-two subjects into two even groups. The averaged global engagement map (0 s delay) over each of the two groups is displayed in Fig. 6 (upper panel). The overall distributions are very similar to one another. The correlation coefficient between the two maps is 0.9111 (95% CI, 0.9099–0.9122). The lower panel of Fig. 6 shows how the reproducibility changes when assuming different delay times. The correlation coefficient remains a relatively high value when assuming 2 s or 4 s delay for WM signal, but drops dramatically to 0.34 when 6 s delay is assumed. We also examined the intra-subject reproducibility based on a publicly available dataset (Salehi et al., 2020) and included the results in the supplementary file (Figure S2). It shows that the intra-subject reproducibility is quite high at 0, 2, and 4 s’ delays, which outperforms the inter-subject reproducibility shown in Fig. 6. Note that our experiments found that WM is barely engaged at a 6 s’ delay, and therefore the engagement map tends to exhibit random patterns, which gives a poor inter- and intra-subjects reproducibility shown in Fig. 6 and S2.

### 3.4. Group-based local functional engagement maps of WM

**Fig. 7.**
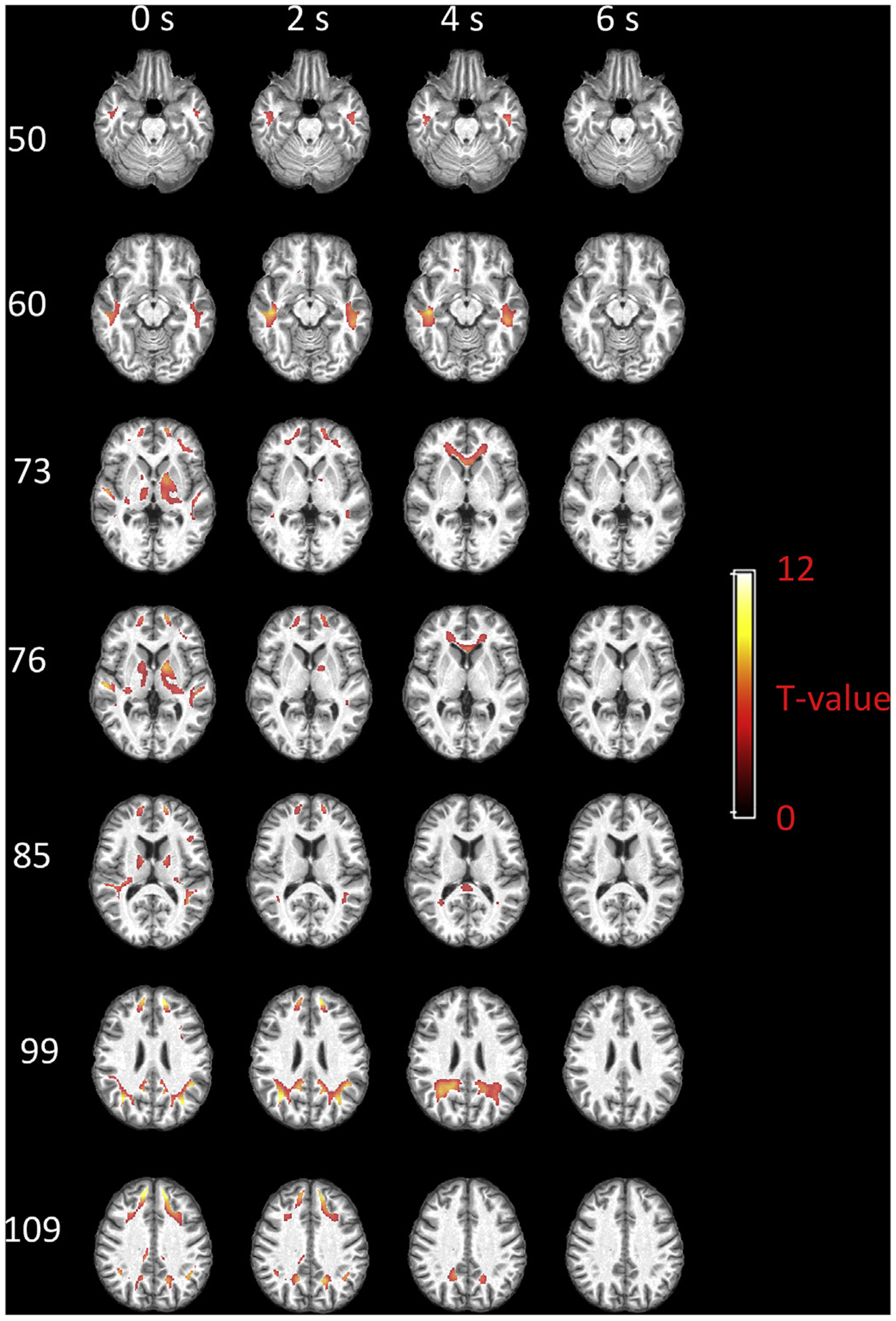
Group-based local functional engagement map of WM with respect to AG. Selected axial slices displayed in rows and the maps regarding four assumptions of WM delays displayed in columns. T values beyond 3.25 (p < 0.01, FDR corrected) are shown.

Fig. 7 displays the group-based local functional engagement maps of WM with respect to AG. The voxels with T-value beyond 3.25 (p < 0.01, FDR corrected) are shown. When assuming a 0 s delay, significantly engaged voxels are distributed in WM areas that are adjacent to superior/middle temporal gyrus, superior/middle frontal gyrus, PCC, putamen and caudate. Particularly, a subcortical area close to AG exhibits the highest engagement. Then the engaged voxels propagate deeper into WM when assuming a 2 s delay for WM, while voxels near deep GM nuclei are rarely engaged. With a 4 s delay, the engaged voxels in frontal lobe extend to anterior corona radiation as well as genu of corpus callosum, while the engaged voxels in the posterior brain expand and reach the splenium of the corpus callosum. When assuming a 6 s delay, no significant engagement can be identified.

**Fig. 8.**
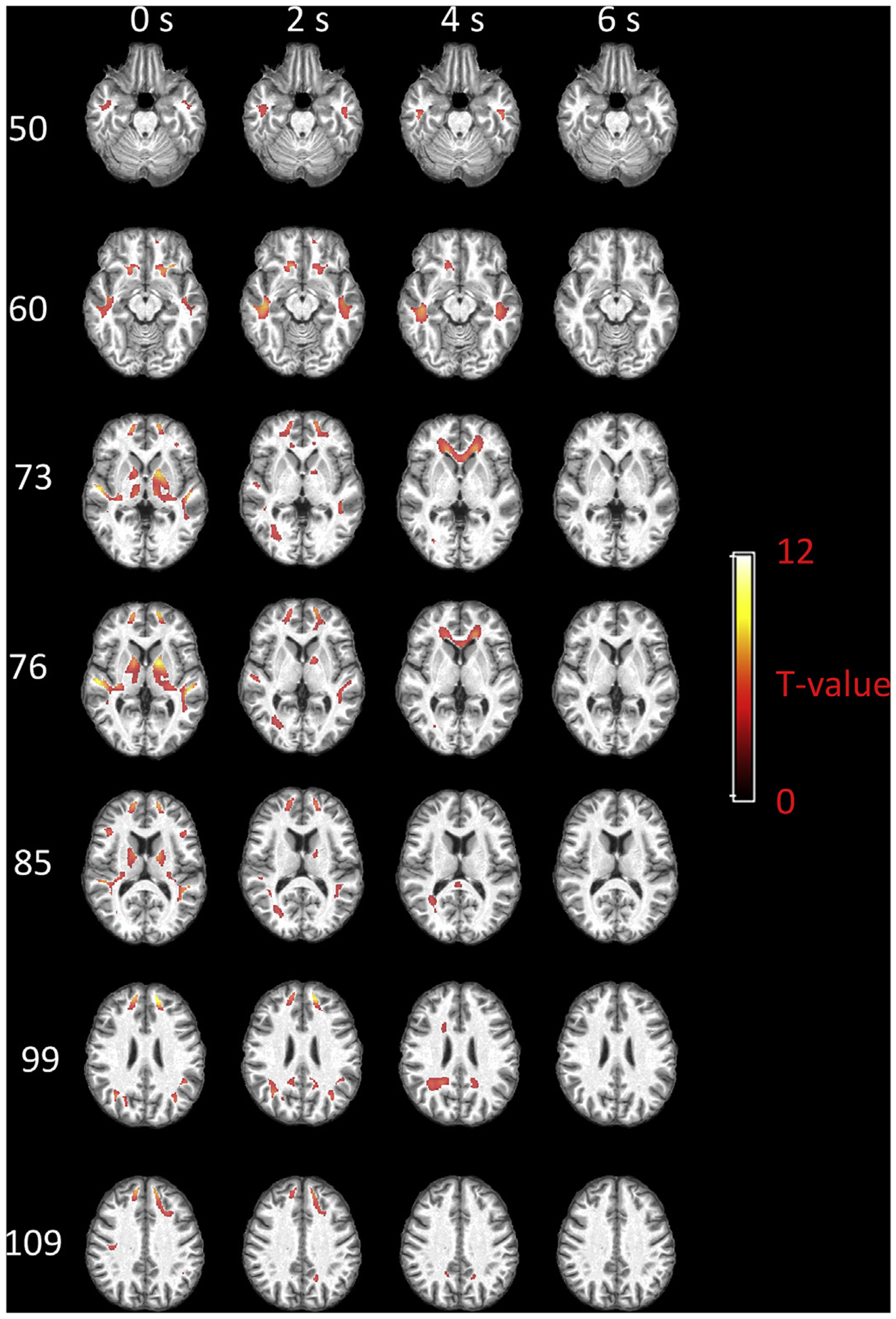
Group-based local functional engagement map of WM with respect to mPFC. Selected axial slices displayed in rows and the maps regarding four assumptions of WM delays displayed in columns. T values beyond 3.25 (p < 0.01, FDR corrected) are shown.

Fig. 8 shows the group-based local functional engagement maps with respect to mPFC. The voxels with T-value beyond 3.25 (p < 0.01, FDR corrected) are shown. Highly engaged WM voxels can be identified within areas neighboring the mPFC, superior temporal gyrus, AG, putamen, caudate and thalamus when a 0 s delay is assumed for WM. These areas expand and reach deeper subcortical WM when assuming a 2 s delay, while voxels near deep GM nuclei are rarely engaged. The engaged voxels further extend to anterior corona radiation, genu of corpus callosum, splenium of corpus callosum and voxels near posterior cingulate gyrus when a 4 s delay is assumed. These engaged voxels disappear when a 6 s delay is assumed.

**Fig. 9.**
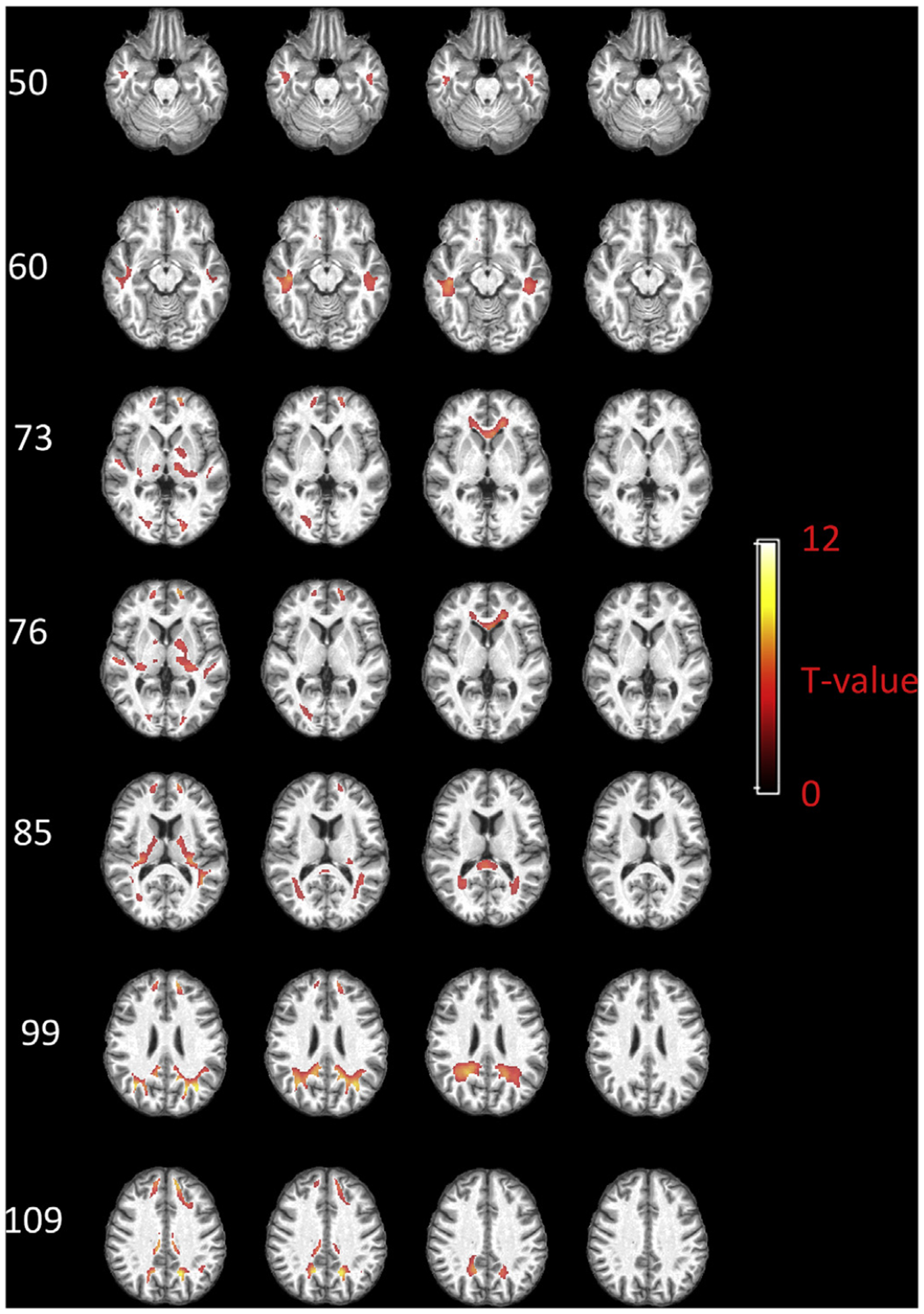
Group-based local functional engagement map of WM with respect to PCC. Selected axial slices displayed in rows and the maps regarding four assumptions of WM delays displayed in columns. T values beyond 3.25 (p < 0.01, FDR corrected) are shown.

Fig. 9 shows the group-based local functional engagement maps with respect to PCC. When 0 s delay is assumed, the important voxels are mainly distributed in areas close to PCC, AG, mPFC, superior/middle temporal gyrus and thalamus. The propagation pattern of these voxels with time (2 s–4 s) is in general similar to that in regards of AG and mPFC. Briefly, they eventually extend to deeper WM area such as genu and splenium of corpus callosum. When assuming a 6 s delay, no engaged voxels survive.

**Fig. 10.**
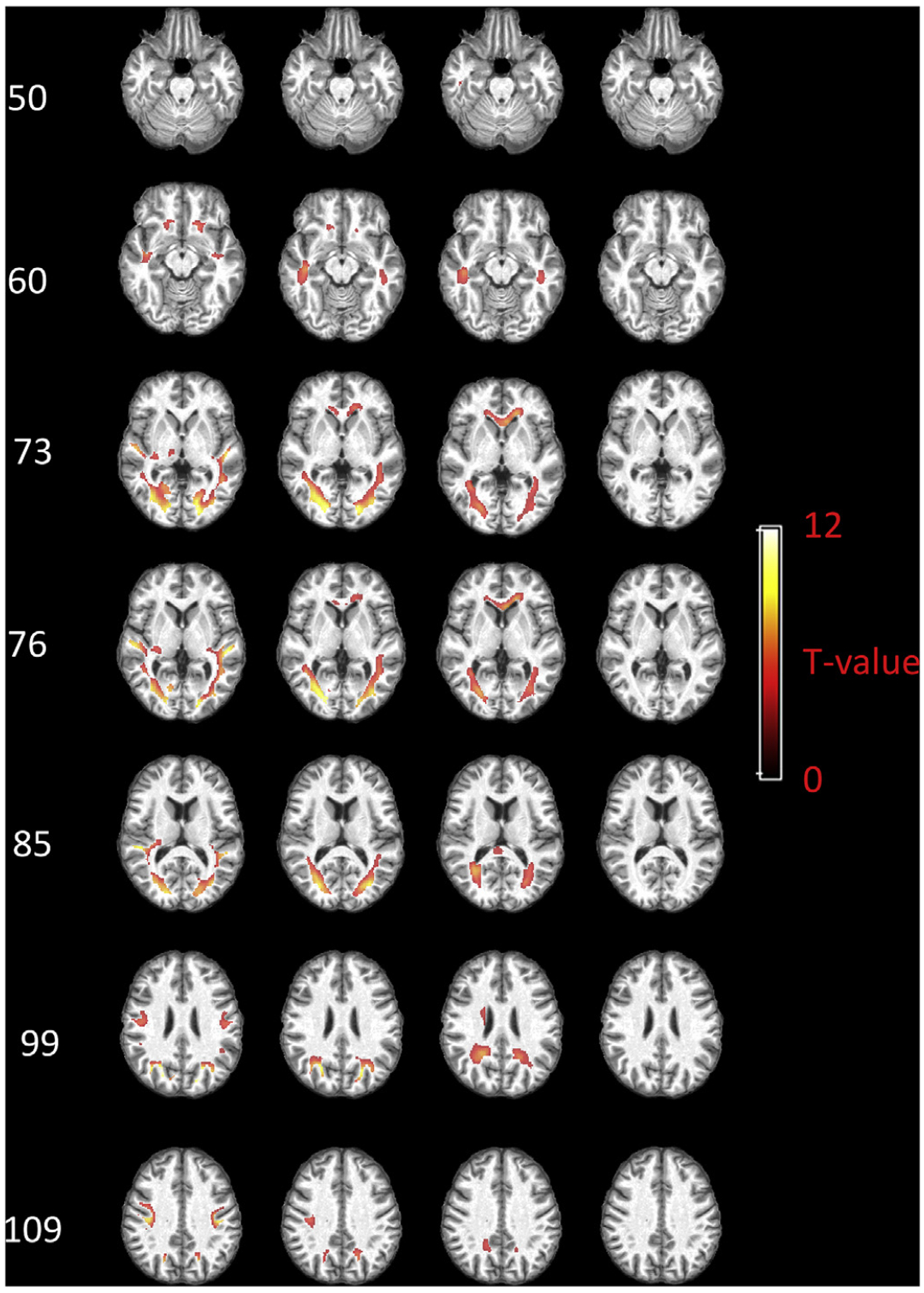
Group-based local functional engagement map of WM with respect to visual cortex. Selected axial slices displayed in rows and the maps regarding four assumptions of WM delays displayed in columns. T values beyond 3.25 (p < 0.01, FDR corrected) are shown.

Fig. 10 shows the group-based local functional engagement map with respect to visual cortex. When 0 s delay is assumed, some voxels neighboring visual cortex, superior temporal gyrus, and pre/post central gyrus are identified with high engagement. Important voxels are subsequently found, in large numbers, along posterior thalamic radiation (including optic radiation), in addition to anterior corona radiation when assuming a 2 s delay. When assuming a 4 s delay, engaged areas extend more deeply, e.g., genu and splenium of corpus callosum. Important voxels are still found in posterior thalamic radiation, but with overall reduced engagement compared with a 2 s delay, and peak values move to deeper WM voxels. When a 6 s delay is assumed, no engaged voxels can be identified.

### 3.5. Variability of local engagement distributions with respect to different GM components

**Fig. 11.**
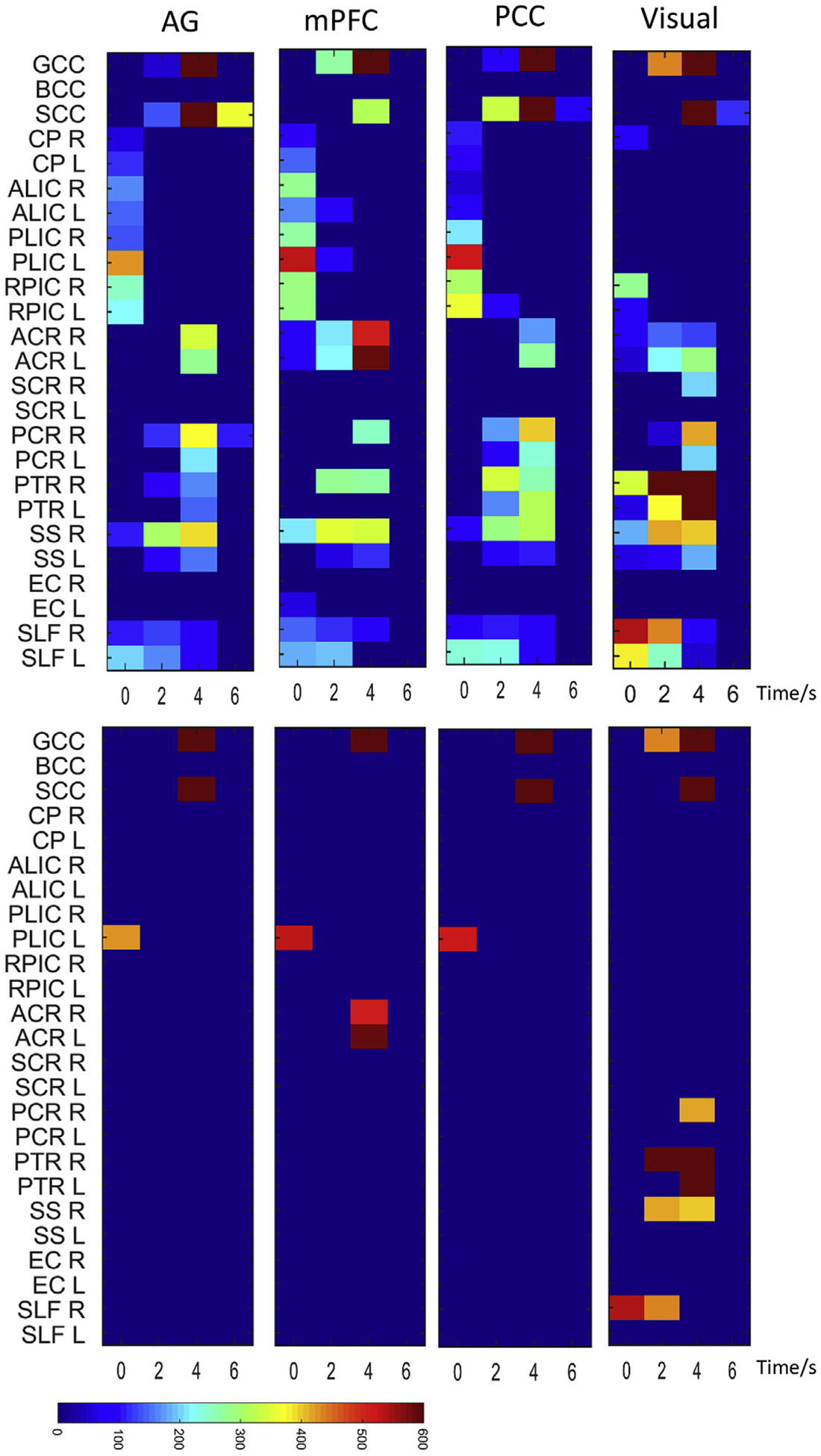
The numbers of significantly engaged voxels in different WM tracts with respect to four local engagement maps. In each sub-panel, the number of significantly engaged (p < 0.01, FDR corrected) voxels that are overlapped with each of 25 WM tracts is plotted in color against the assumed delay time. Upper panels: voxel numbers beyond 50 are shown. Lower panels: voxel numbers beyond 400 are shown.

The numbers of group-based local engaged voxels (p < 0.01, FDR corrected) in each WM structure (tract) that contribute to the connectivity of four different GM nodes are plotted against the assumed delay time in Fig. 11. For AG, mPFC and PCC, the overall patterns are similar to one another, where the maximum numbers of engaged voxels are, overall, identified in corpus callosum when assuming a 4 s delay. For visual cortex, in contrast, the maximum number is recognized in posterior thalamic radiation when assuming a 2 or 4 s delay.

### 3.6. Comparison between global functional engagement and fALFF

**Fig. 12.**
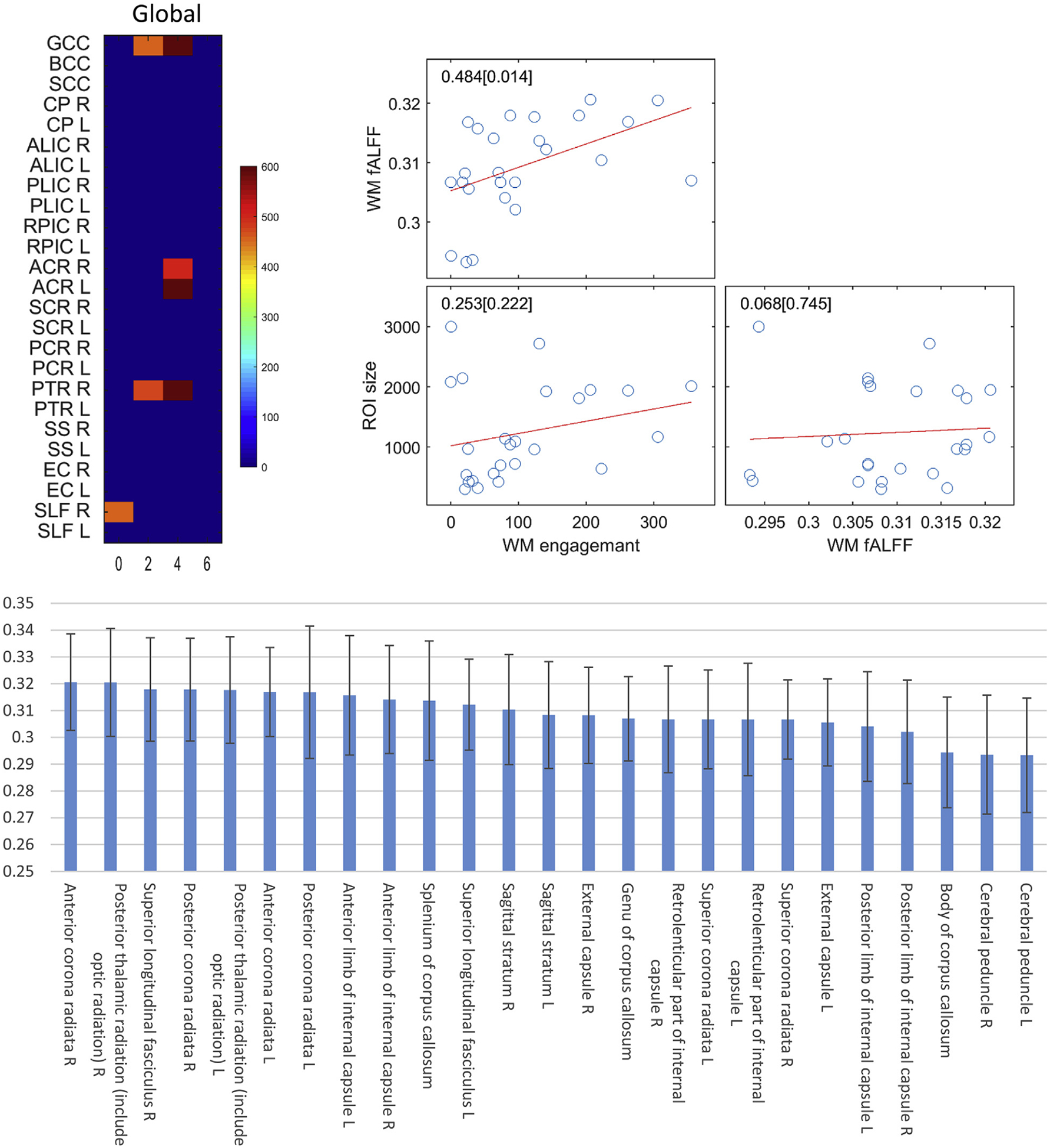
Comparison between global functional engagement and fALFF in 25 selected WM tracts. Upper left panel: the number of significantly engaged (p < 0.01, FDR corrected) voxels that are overlapped with each of 25 WM tracts is plotted in color against the assumed delay time (voxel numbers beyond 400 are shown). Lower panel: fALFF values in 25 WM tracts ranked in descend order. Each bar is the fALFF averaged across the population and is overlaid a 1SD. Upper right panel: scatter plot of WM engagement against fALFF and ROI size of 25 selected WM tracts.

At a global level, the number of significantly engaged (p < 0.01, FDR corrected) voxels that overlap with each of 25 WM tracts is plotted in color against the assumed delay time in the upper left panel of Fig. 12. The top five engaged tracts are genu of corpus callosum, anterior coronal radiation left, anterior coronal radiation right, posterior thalamic radiation right and superior longitudinal fasciculus right. The lower panel of Fig. 12 shows the fALFF of 25 tracts, sorted in descending order. It can be observed that highly engaged tracts, including anterior coronal radiation right, posterior thalamic radiation right and superior longitudinal fasciculus right, are also ranked in the top three with regards to their fALFFs. The results of correlation among WM fALFF, WM engagement and ROI sizes are shown in the upper right panel of Fig. 12, which indicates that fALFF is significantly correlated with WM engagement (r = 0.484, p = 0.014) across ROIs. Meanwhile, no significant correlation can be identified between ROI size and fALFF/engagement.

## 4. Discussion

We have defined a novel measure of WM voxels, namely, WM engagement, which quantifies their functional importance to networks of GM nodes. We observe a distinct and highly reproducible distribution of global WM engagement during a resting state. This distribution varies both spatially and temporally in that, overall, important WM voxels tend to propagate from superficial to deep WM with an increasing time delay (maximum 6 s). This finding is consistent with the neurovascular anatomy, according to which, deeper WM areas are more distant from their feeding arterioles, requiring longer times for them to receive increased blood supply (Thomas et al., 2014). Regardless of the time delay, the most engaged voxels at a global level are distributed in the anterior coronal radiation, posterior thalamic radiation, genu and superior longitudinal fasciculus. Most of these areas are consistent with WM bundles that show the highest fALFF during a resting state. In GM networks, fALFF is interpreted as a measure of spontaneous neural activity and is highest in the main components of the default mode network, including PCC, mPFC and inferior parietal lobule (IPL) (Yu-Feng et al., 2007; Zou et al., 2008), which have also been consistently reported to be the network hubs in the human brain (Buckner et al., 2008; Jonathan D Power et al., 2013; van den Heuvel and Sporns, 2013). Analogous to these GM hubs, the WM bundles with higher spontaneous intrinsic BOLD signal fluctuations appear to contribute most to the network connectivity, potentially acting as highly engaged paths that interconnect a larger number of segregated functional nodes. By using a tractography approach based on diffusion images acquired from the same subjects, we explore the structural connection between highly engaged voxels in deep WM, i.e., the ones shown in the third column of Fig. 4, and the cortex. The results are included in the supplementary file (Figure S4). Structural connections can be identified from the seeding regions to the ventromedial prefrontal cortex (mPFC), anterior cingulate cortex (ACC), supplementary motor area (SMA), posterior cingulate cortex (PCC), middle temporal area, middle/superior occipital areas, superior parietal areas as well as angular gyrus. Most of these areas are components of important brain networks such as the default mode network, visual network, and supplementary motor network, thereby certifying the important roles of highly engaged WM voxels.

The spatial distributions, as well as the temporal propagation patterns of local engagement maps relevant to AG, mPFC and PCC are, in general, consistent with one another, exhibiting high values in corpus callosum and anterior coronal radiations. A possible explanation is that AG, mPFC and PCC are all major components of the DMN, and as such exhibit highly correlated inter-regional BOLD fluctuations. When regressing out the signal from a selected WM voxel, each part of the network may undergo a comparable reduction of local connectivity to the other GM regions, so that the local engagement of that WM voxel is similar for each node. The local engagement map with respect to visual cortex, in contrast, exhibits the highest values in posterior thalamic radiation (including optic radiation) regardless of time delay. The differences in spatial distribution possibly attribute to a notion, which has been demonstrated in an animal model by our previous work (Wu et al., 2019), that BOLD correlations between WM tracts and GM areas are related to their anatomical connections.

A potential confound is that white matter BOLD signals may arise secondary to gray matter functional activity because of venous drainage effects. However, according to the venous system configuration reported by Pearl et al. (2011), there are two separate venous systems for GM and WM: a superficial venous system that drains deoxygenated blood in the cortex and superficial WM into venous sinuses via cortical veins, and a deep venous system that drains deoxygenated blood in deep WM into the subependymal veins. As such, there are minimal vascular interactions between the two different tissue types, and the blood flowing out of activated cortical regions is unable to reach deep WM to modulate the signals therein (exceptions exist for developmental venous anomalies but these have a maximum incidence of only 2.6% (Pearl et al., 2011)). The superficial venous system does also drain blood from subcortical white matter. However, according to the same paper, it drains in a centrifugal direction from WM to the cortex and then to pial veins. Thus effects in WM are less likely to suffer directly from the BOLD changes detected in cortical regions. Even so, in this study, we made our best effort to make sure superficial WM voxels were not included in the WM mask by setting a very high threshold (0.95) to eliminate partial volume effects. To better understand the origin of the WM engagement, we repeated the whole process for GM and included the results in the supplementary file (Figure S4). As can be seen in Figure S4, most significantly engaged WM areas are not extensions of engaged GM nearby, indicating that signals in these WM areas are not simply from contaminations by adjacent GM. Note that the phenomenon is most pronounced at a time delay of 4s. To sum up, WM BOLD signals are not simply a secondary effect caused from GM activity via vascular system. Another confound is that WM activities might be attributed to physiological fluctuations and head movement. To examine this, we perform a voxel-wise analysis of temporal correlations of WM signals with the physiological measures as well as head movement parameters we obtained. The voxels that highly correlate with these covariates are found to have little overlap with the engaged WM voxels. Given that these covariates have been regressed out from WM time series prior to our engagement analysis, it can be essentially ruled out that the WM engagement map derived in this study is largely leveraged by physiological fluctuations or the head movement.

The GM nodes that strongly contribute to network functions are referred to as cortical hubs, the breakdown of which can cause rearrangement of the network’s topology and lead to dramatically altered global metrics. Functional couplings of hubs have been reported in many studies (e.g. 34), suggesting their relationships to individual differences and development, for instance, intelligence (Hearne et al., 2016) and age (Jones et al., 2011), in addition to their contributions to brain disorders, such as schizophrenia (Rubinov and Bullmore, 2013), autism (Padmanabhan et al., 2017) and Alzheimer’s disease (Dai et al., 2015). In a similar vein, the engagement maps described in this study may also be used as a filter to identify WM regions that play critical roles in the integration and communication between GM nodes in the brain. Highly engaged WM areas can be further analyzed to extract fMRI-based measurements, including Amplitude of Low-Frequency Fluctuations (ALFF), Regional Homogeneity (ReHo), Degree Centrality (DC) and WM engagement itself, from patients with neurological or psychiatric disorders, as well as normal controls. With the suite of measurements, functional alterations in the engaged areas can be quantitatively explored on a regional basis to determine imaging markers of such diseases, as well as their relationships to clinical parameters including age, gender, behavior assessments and other imaging biomarkers measurable from structural MRIs (e.g., atrophy of cortical structures and impairment of WM integrity). Moreover, it enables investigations of waxing, waning or shifting of highly engaged WM voxels during development, aging or neuro-degeneration. These goals have been fundamental drivers of neuro-imaging research, and have conventionally relied on the detection of functional activities in GM by fMRI and identification of structural organization of WM by diffusion tensor imaging (Basser et al., 1994). This conventional separation, however, may not be able to faithfully represent structure-function relationships in the brain. By employing only BOLD contrast for both GM and WM, and aided by the WM time-dependent engagement maps as shown in this work, improved knowledge of the functional architecture may be gained.

There are four limitations of the current work. First, fMRI cannot offer high enough temporal resolution for directly modeling the timings of neural events, which is typically of the order of milliseconds and requires regional knowledge regarding vascular distribution and properties. However, these studies further suggest that BOLD signals in WM reflect neural activities, and their removal in typical studies that aim to depict neural networks is inappropriate. Second, even though we applied a tight threshold to probabilistic WM masks to ensure the engagement map being limited to within WM, GM contaminations may not be completely excluded due to potential mis-registrations between fMRI and T1 caused by EPI distortions during imaging. Therefore, the engagement of some superficial WM regions might be attributable to contributions from adjacent GM that has been misclassified as WM due to image distortions. In our future studies B0 field mapping will be implemented for distortion corrections to allow more accurate interpretations. Next, for the simplicity of computations, in this work, we use the difference between the sum of a full correlation matrix and that of a partial correlation matrix to quantify the engagement of a WM voxel. Obviously, this simple approach comes with certain limitations. First of all, it does not consider the polarity of functional connectivity. As such, increases in negative connectivity values might be taken as decreases in connectivity and thus engagement measure. In addition, the pattern change of the matrices is not captured, e.g., matrix [3 2 1] and [1 2 3] are treated as zero difference. To ameliorate these problems, in future studies, we will examine various metrics such as mean square errors, or differences of advanced graph-theoretical parameters that have been frequently reported to be relevant to functional measures, so that both the magnitude and pattern changes in correlation matrices are characterized. Last, we would like to point out that participants with potential risk factors for white matter disease, such as cigarettes smoking, aging, cardiovascular disease, high blood pressure and high cholesterol, were not excluded from the recruitment process and thereby could bias interpretations of our findings. These potential risks for white matter disease will be meticulously considered in our future studies to ensure more accurate repretentaions of WM engagement for the healthy population.

In conclusion, we investigated the engagement of WM contributions to the functional connectivity of networks in the brain using a partial correlation approach. The measured global engagement maps are highly reproducible and trend toward increased engagement values of deeper WM over time. The local engagement exhibits homogenous spatial distributions regarding internal nodes that constitute a sub-functional network, but inhomogeneous distributions regarding other nodes. Taken together, these findings demonstrate the important roles of WM in the couplings within functional networks and emphasize the need to consider WM fluctuations in the analysis of resting state studies.

## Supplementary Material

1
