# Supplementary material for: Functional engagement of white matter in resting-state brain networks
Source: Neuroimage. Author manuscript; Available in PMC 2020 Oct 29. (PMC7594260; doi:10.1016/j.neuroimage.2020.117096)
Supplement: 1 [file NIHMS1639063-supplement-1.docx]

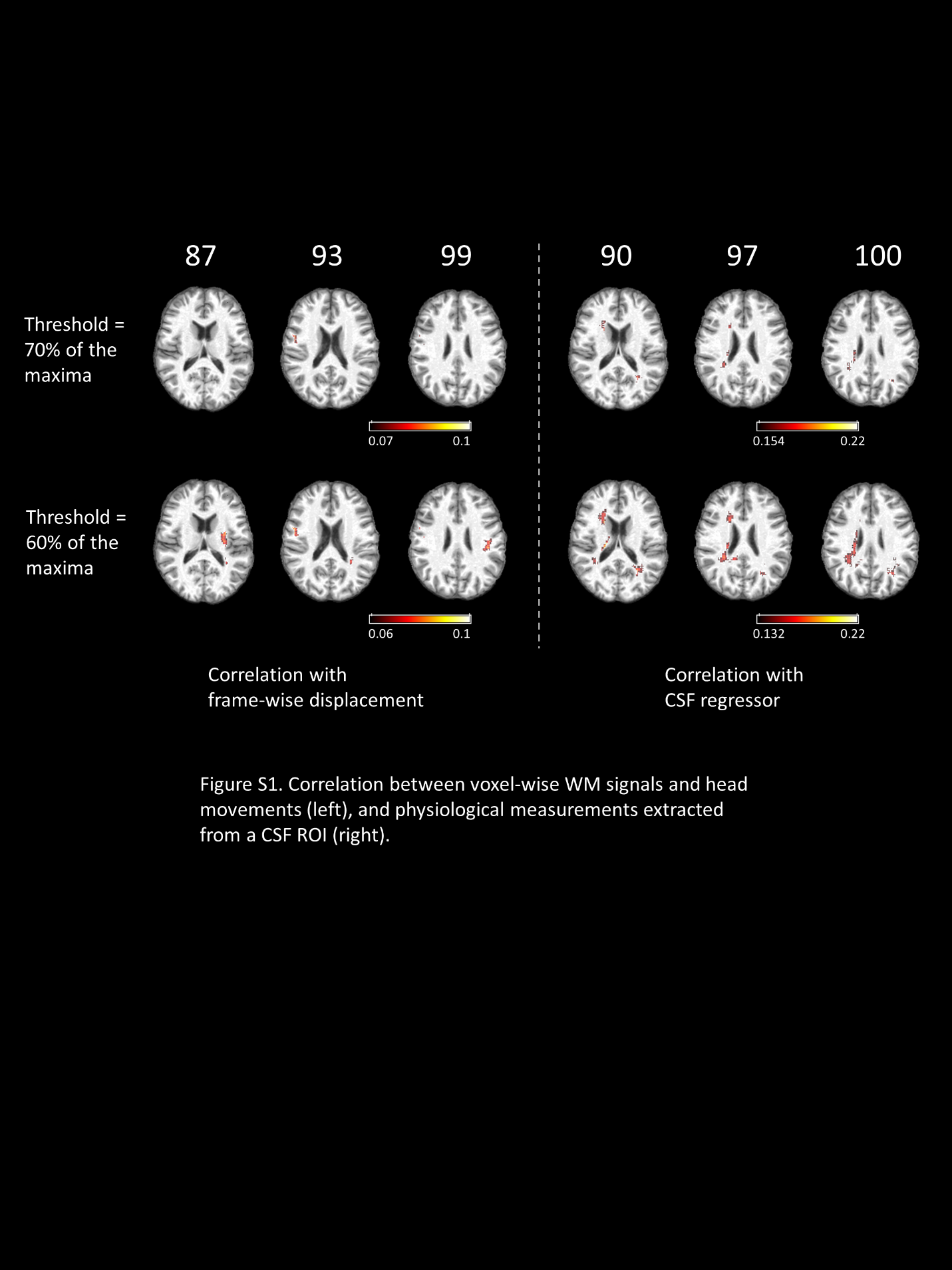


Figure S1. Correlation between voxel-wise WM signals and head movements (left), and physiological measurements extracted from a CSF ROI (right). The voxels that highly correlate with head movement (framewise displacement, FD) are mainly distributed in areas close to the bilateral postcentral cortex and right external capsule. The voxels exhibit relatively higher correlate with physiological measurement are mainly distributed in areas close to lateral ventricles.


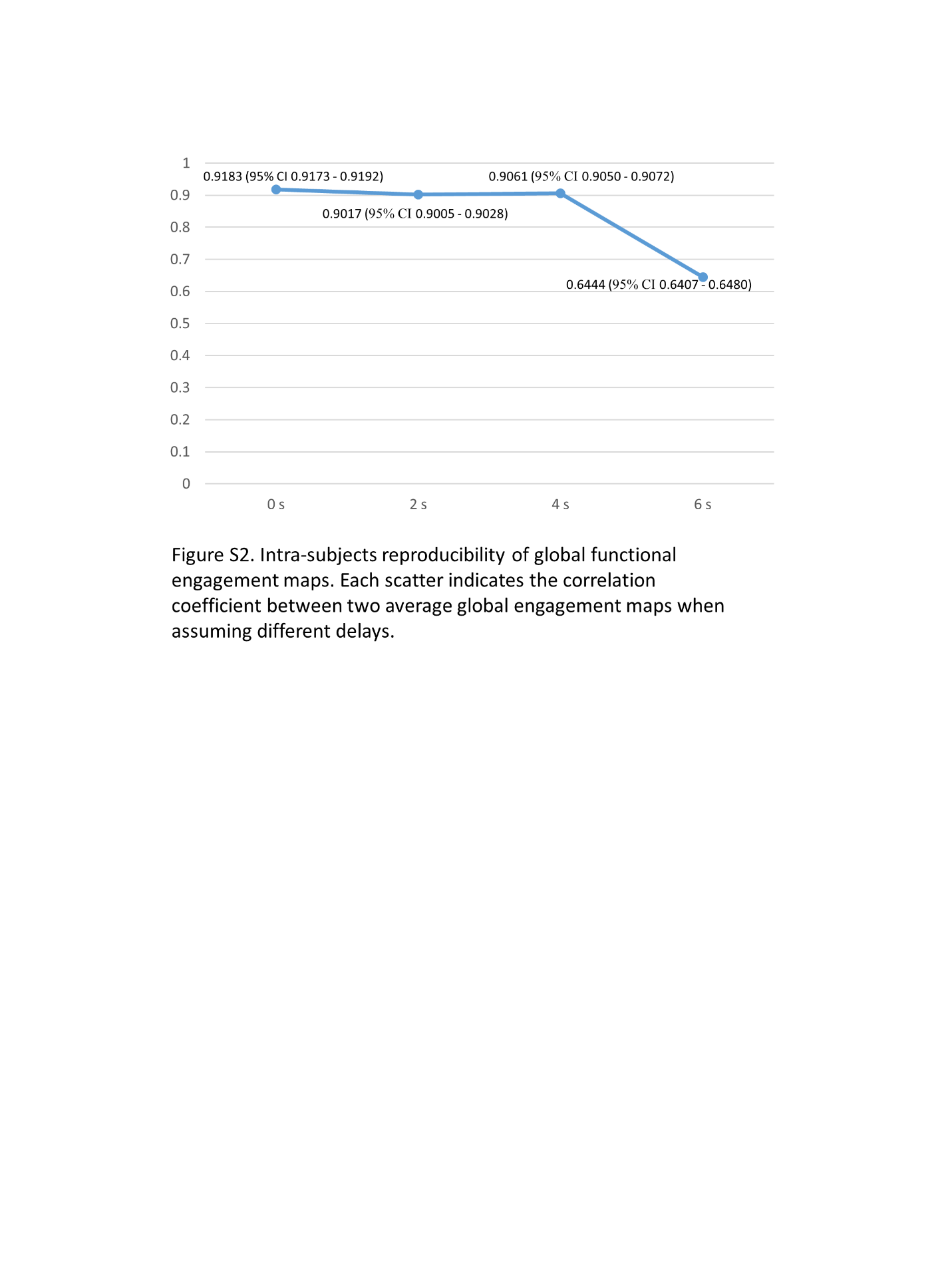


Figure S2. Intra-subject reproducibility of global functional engagement maps. Each scatter indicates the correlation coefficient between two average global engagement maps when assuming different delays.

This experiment is based on a publicly available dataset, namely, Yale Single Subject Task Rest 30X (<https://openneuro.org/datasets/ds002372/versions/1.0.0>), which consists of 30 sessions of fMRI data. We were able to identify one subject of 56 years male, from who two resting-state runs (run1 and run2) were acquired in each of the 30 sessions. We averaged the run1 and run2 data separately across the 30 sessions and computed the Pearson’s correlation between the two averaged engagement maps.


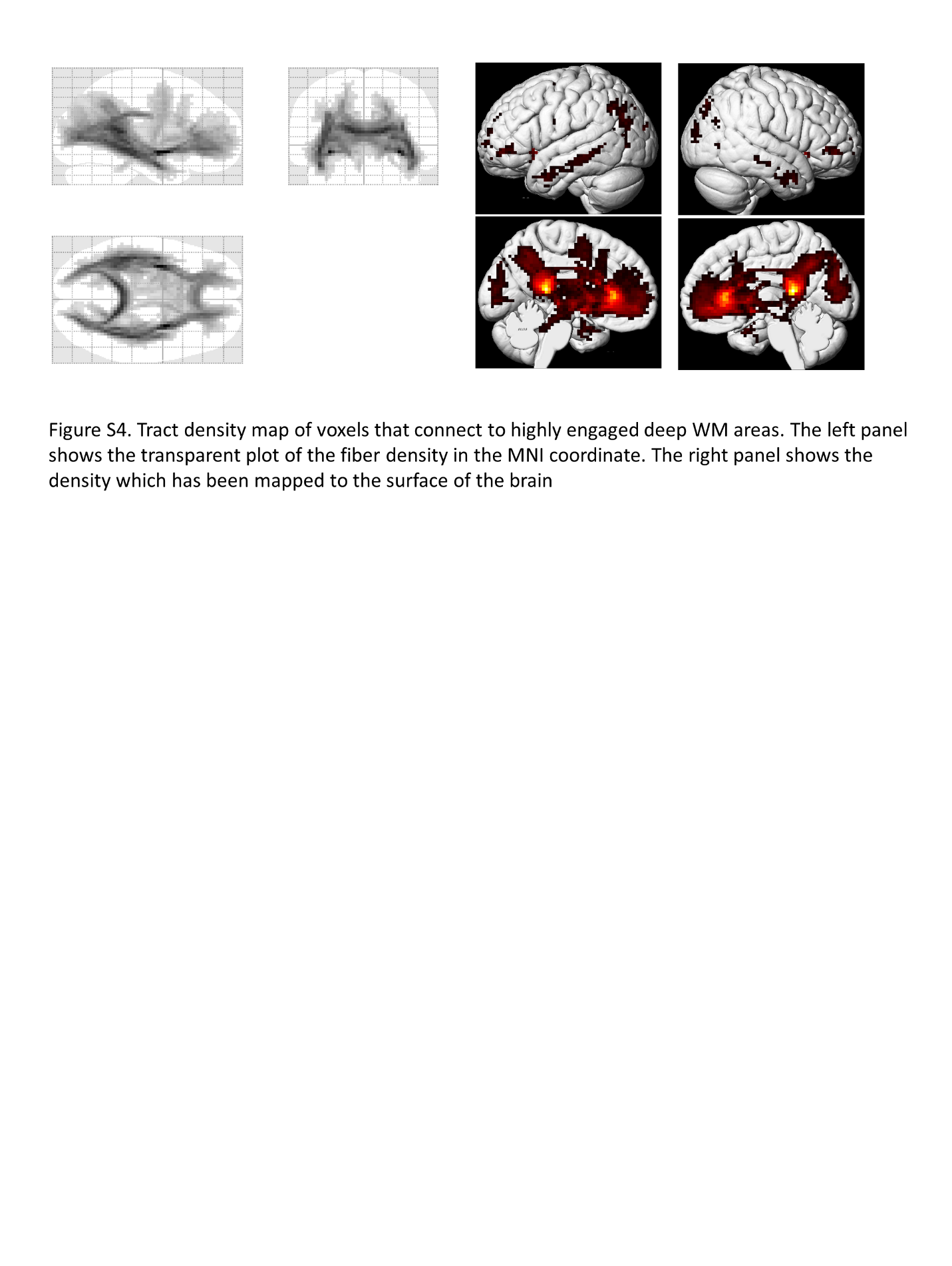


Figure S3. Tract density map of voxels that connect to highly engaged deep WM areas. The left panel shows the transparent plot of the fiber density in the MNI coordinate. The right panel shows the density on the surface of the brain.

We performed diffusion-based tractography by considering highly engaged voxels in deep WM, i.e., the ones shown in the 3rd column of Figure 4, as the seeding regions. Diffusion images were acquired using a multi-shot, echo-planar imaging (EPI) sequence with b = 1000 s per mm2, 32 diffusion-sensitizing directions, TR = 4.5 s, TE = 84 ms, matrix size = 112 × 112 × 68, and voxel size = 2 × 2 × 2 mm3. For each individual, the diffusion model was reconstructed in the MNI space using q-space diffeomorphic reconstruction (QSDR) algorithm from DSI Studio (<http://dsi-studio.labsolver.org/>). Then a deterministic fiber tracking algorithm was used to search throughout the rest of the brain for all possible voxels that connect to the seeds. The approach yields a tract density map in which the value of density reflects the possibility that WM fibers traverse the location. A population-based density map is then computed by averaging across all the 53 subjects studied. The left panel shows transparent renderings of the fiber density in the MNI space. The right panel shows the density on the surface of the brain, which exhibits connection from the seeding regions to the ventromedial prefrontal cortex (mPFC), anterior cingulate cortex (ACC), supplementary motor area (SMA), posterior cingulate cortex (PCC), middle temporal area, middle/superior occipital areas, superior parietal areas as well as angular gyrus. Most of these areas are components of important brain networks such as the default mode network, visual network, and supplementary motor network.


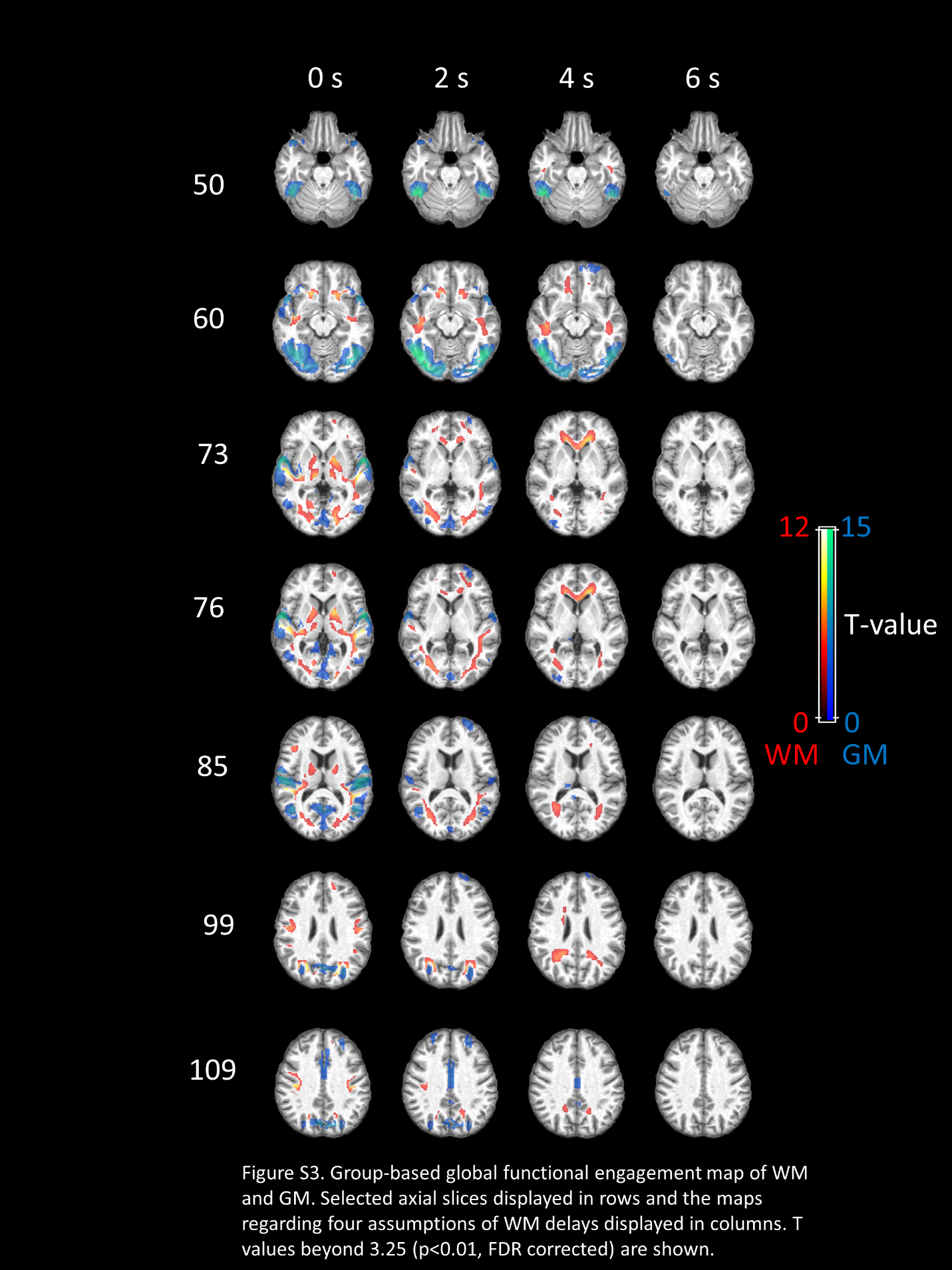


Figure S4. Group-based global functional engagement map of WM and GM. Selected axial slices displayed in rows and the maps regarding four assumptions of WM delays displayed in columns. T values beyond 3.25 (p<0.01, FDR corrected) are shown. GM voxels were analyzed using the same pipeline that was for WM but was limited in a binarized (>0.6) population-averaged GM mask.
